# Schlaganfallpfad Tirol

**DOI:** 10.1007/s10049-022-01016-w

**Published:** 2022-04-15

**Authors:** Christian Boehme, Stefan Krebs, Theresa Geley, Heinrich Rinner, Andreas Maurer, Julia Runge, Johannes Schoech, Johann Willeit, Stefan Kiechl, Michael Knoflach

**Affiliations:** 1 Universitätsklinik für Neurologie, Medizinische Universität Innsbruck, Anichstr. 35, 6020 Innsbruck, Österreich; 2 Abteilung für Neurologie, Krankenhaus der Barmherzigen Brüder, Wien, Österreich; 3 Tiroler Gesundheitsfonds, Innsbruck, Österreich; 4 Leitstelle Tirol gemeinnützige GmbH, Innsbruck, Österreich; 5 Landesinstitut für Integrierte Versorgung Tirol, Innsbruck, Österreich

**Keywords:** Notfalltherapie/Schlaganfall, Behandlungspfad, Rettungskette, Versorgungsqualität, Prähospitale Notfallversorgung, Emergency treatment/stroke, Care pathway, Rescue chain, Quality of health care, Prehospital emergency care

## Abstract

**Hintergrund:**

In der Behandlung des Schlaganfalls gibt es Fortschritte auf vielen Ebenen. Dies führt zu besseren Heilungschancen und einer Minderung körperlicher Beeinträchtigung. Akuttherapiemaßnahmen sind auf ein kurzes Zeitfenster nach Auftreten limitiert, deshalb ist das Notfallmanagement besonders kritisch. Das Projekt Schlaganfallpfad Tirol wurde realisiert, um die Versorgungskette beim Krankheitsbild Schlaganfall von der prähospitalen Phase bis zum Abschluss der Rehabilitation zu optimieren.

**Ziel der Arbeit:**

Beschreibung des Tiroler Schlaganfallpfads als Beispiel für die Optimierung der Schlaganfallversorgung in einer mitteleuropäischen alpinen Region mit Schwerpunkt auf die prähospitale Versorgung.

**Material und Methoden:**

In vier Teilprojekten von der Prähospitalphase bis zur Nachbehandlung wurden Versorgungsprozesse und Schnittstellen optimiert und mit Qualitätssicherungsmaßnahmen evaluiert.

**Ergebnisse:**

Nach Implementierung hat sich die Thrombolyserate fast verdoppelt und die Rate an gutem funktionellem Outcome nach 3 Monaten verbessert. Komplikationen wie eine Aspirationspneumonie haben deutlich abgenommen. Der Zugang zu rehabilitativen Maßnahmen hat sich verbessert, insbesondere auch wegen des Aufbaus einer qualitätskontrollierten und finanzierten ambulanten Rehabilitation.

**Schlussfolgerung:**

Ein ganzheitliches Versorgungsprojekt kann gut in die Praxis umgesetzt werden und verbessert die Versorgungsqualität beim ischämischen Schlaganfall. Der europäische Aktionsplan Schlaganfall 2018–2030 empfiehlt die Etablierung umfassender Schlaganfallpfade in allen Regionen und Ländern Europas.

## Abstract (en)

**Background:**

Recent advances in acute stroke therapy have been shown to improve clinical outcome and therefore reduce long-term disability. Acute therapy procedures in stroke have a limited time frame after onset of symptoms; thus, rapid emergency management is critical. The Tyrolean Stroke Pathway was developed to optimize the entire treatment pathway from stroke onset to outpatient rehabilitation.

**Objectives:**

The description of the Tyrolean Stroke Pathway as an example to optimize the entire treatment pathway of stroke patients in a central European alpine region with a focus on prehospital management.

**Materials and methods:**

In four subprojects from the prehospital phase up to outpatient rehabilitation, the project optimized intersections between different phases of care and evaluated the care processes using quality assurance measures.

**Results:**

After implementation, thrombolysis rates doubled and functional outcome of stroke patients after 3 months improved and complications, e.g., pneumonia, declined. Access to rehabilitation improved, particularly because of the establishment of quality assured and funded outpatient rehabilitation.

**Conclusions:**

The project showed that a holistic standardized stroke pathway from acute management up to outpatient rehabilitation can be successfully implemented in clinical routine. The project improved the entire health care pathway of stroke patients. The Stroke Action Plan for Europe 2018–2030 recommends to have national plans for stroke that encompass the entire chain of care.

Um die Versorgungskette beim Schlaganfall vom Erkennen der Erkrankung bis hin zum Abschluss der ambulanten Rehabilitation zu optimieren, wurde der Tiroler Schlaganfallpfad realisiert. Das standardisierte und qualitätsgesicherte Versorgungskonzept schließt alle beteiligten Berufsgruppen ein und hinterlegt Prozessabläufe für alle Behandlungsschritte.

Der Schlaganfall stellt nach Tumoren und Myokardinfarkt die dritthäufigste Todesursache in Österreich dar, er ist die häufigste Ursache von körperlicher Behinderung im Erwachsenenalter und eine häufige Ursache für die Entwicklung einer Demenz [1]. Durch Fortschritte in der Akuttherapie, besonders durch die Einführung der Thrombolyse und Thrombektomie, werden bessere Heilungschancen und eine Minderung körperlicher Behinderung erreicht.

Um die Versorgungskette beim Krankheitsbild Schlaganfall zu optimieren, hat das Land Tirol gemeinsam mit den Versicherungsträgern im Jahr 2006 das Projekt „Integrierter Patientenpfad/Behandlungspfad Schlaganfall“ ins Leben gerufen. Ziel des Projekts ist es, ein standardisiertes ganzheitliches Konzept zu etablieren, das die gesamte Schlaganfallversorgungskette optimiert und eine stärkere Vernetzung aller beteiligten Berufsgruppen (Allgemeinmediziner*innen, Fachärzt*innen, Therapeut*innen, Pflegepersonal etc.) und Sektoren (Rettungswesen, Krankenhäuser, Pflegeeinrichtungen, mobile Pflege, Rehabilitationseinrichtungen, Sozialversicherungsträger) fördert. Ziel ist es, eine Verbesserung der Versorgungsqualität beim Schlaganfall zu erreichen.

## Situation im Bundesland Tirol (Österreich)

**Figure 1:**
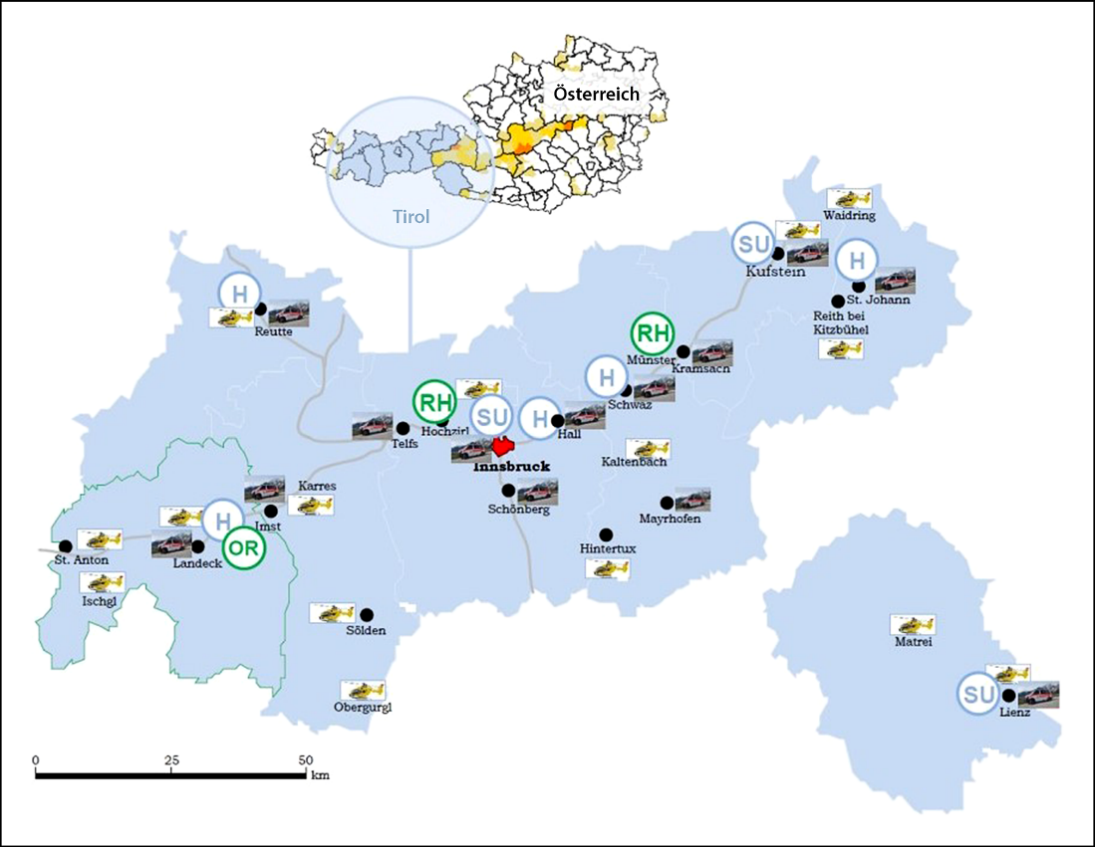

Der Bevölkerung von Tirol (750.000 Einwohner*innen) sowie zahlreichen Tourist*innen mit jährlich > 45 Mio. Übernachtungen stehen acht öffentliche Krankenanstalten und drei öffentliche Sonderkrankenanstalten mit rund 4000 Betten zur Verfügung. Patient*innen mit akutem Schlaganfall werden in allen Akutkrankenhäusern auf neurologischen und internistischen Abteilungen behandelt. Spezialisierte Stroke Units sind an der Universitätsklinik Innsbruck sowie an den Bezirkskrankenhäusern Kufstein und Lienz verfügbar. Aufgrund der geografischen Gegebenheiten mit Haupt- und vielen Seitentälern und dem gebirgigen Landschaftsbild ist bei allen medizinischen Notfällen der zeitnahe Rettungstransport in das dafür geeignete Krankenhaus oft eine Herausforderung. Um dieser Herausforderung zu entsprechen, stehen in Tirol 13 bodengebundene Notarztrettungsmittel und bis zu 16 Notarzthubschrauber für die Disposition durch die Leitstelle Tirol zur Verfügung. Sie ermöglichen es, fast jeden Ort in Tirol in weniger als 15 min zu erreichen (Abb. 1).

## Überblick über den Schlaganfallpfad Tirol

**Figure 2:**
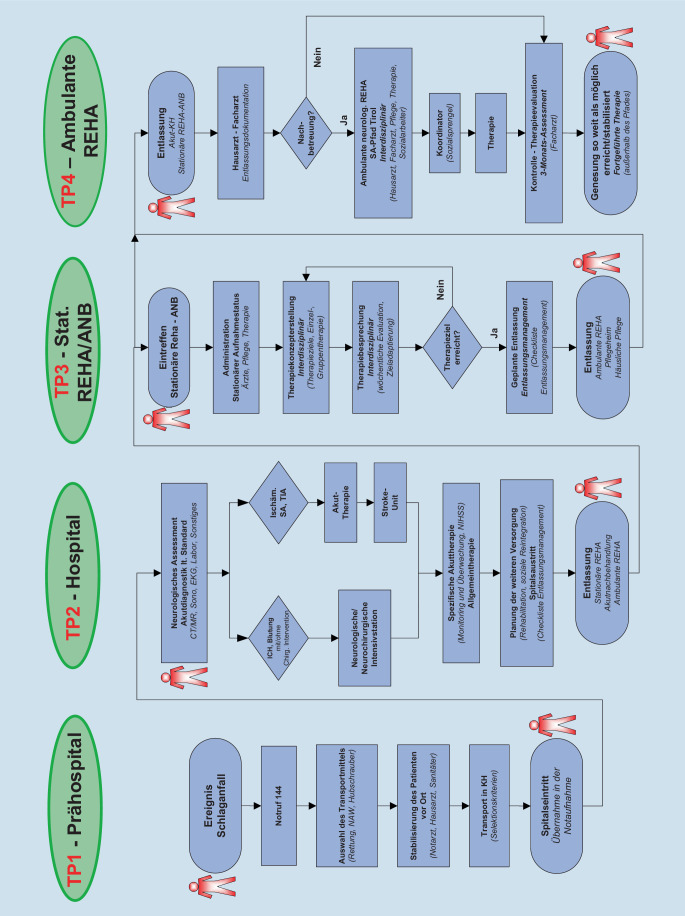

Nach zweijähriger Planungsphase wurden die Kernelemente des Tiroler Schlaganfallpfads 2009 aktiv geschaltet und werden seither kontinuierlich angepasst und erweitert. Mittlerweile wurden mehr als 10.000 Schlaganfallpatient*innen dem Pfad entsprechend behandelt. Seit 2020 ist nun auch die ambulante Rehabilitation zu Hause flächendeckend umgesetzt. Um alle Aspekte der Schlaganfallversorgung sowie die Nahtstellen und Übergänge zwischen den einzelnen Phasen der Versorgung optimieren zu können, wurde die Organisation auf vier Teilprojekte aufgeteilt. Zu den Projektbeteiligten gehören der Tiroler Gesundheitsfonds mit seinen Fondskrankenanstalten, die Abteilung Soziales des Landes Tirol, die Tiroler Sozialversicherungsträger, die Tirol Kliniken und Bezirkskrankenhäuser, das Landesinstitut für Integrierte Versorgung und die Ärztekammer. Neben einer ärztlichen Leitung des Projekts (Kiechl, Willeit) wurde auch ein wissenschaftlicher Beirat installiert und für jedes der vier Teilprojekte ein eigenes Projektteam gebildet (Abb. 2).

Der Tiroler Schlaganfallpfad ist online in allen Krankenanstalten hinterlegt. Mittels eines interaktiven Dokuments können Details zu den Prozessabläufen der einzelnen Teilprojekte aufgerufen und die entsprechenden Arbeitshilfen und Standardarbeitsanweisungen heruntergeladen werden, die sich an den internationalen und nationalen Behandlungsrichtlinien orientieren und für die regionale Situation angepasst sind.

Für den Erfolg des Projekts sind neben der Involvierung aller beteiligten Berufsgruppen das zentrale und erfahrene Projektmanagement des Tiroler Gesundheitsfonds sowie die Installierung einer Qualitätssicherung mit klaren Qualitätskriterien verantwortlich.

### 1. Teilprojekt – Prähospitalphase

In der Akutphase eines Schlaganfalls ist sowohl das Erkennen von Schlaganfallsymptomen durch den Laien bzw. durch Notrufexpert*innen der Rettungsleitstelle als auch eine reibungslos funktionierende Rettungskette notwendig, da die Zeit vom Auftreten von Symptomen bis zur Behandlung eine zentrale Rolle spielt. Die Ausschöpfung der spezifischen Akuttherapie ist nur in den ersten Stunden nach Auftreten eines Schlaganfalls möglich, und mit jeder Minute, in der das Gehirn mit Blut unterversorgt ist, sinkt die Wahrscheinlichkeit einer guten Erholung („Time is brain“). Deshalb ist das Notfallmanagement bei Schlaganfallpatient*innen besonders kritisch. Auf die Prähospitalphase wird unten im Detail eingegangen.

### 2. Teilprojekt – Hospitalphase

Bei Eintreffen im Krankenhaus sichert eine möglichst rasch durchzuführende zerebrale Bildgebung (Computertomographie/Magnetresonanztomographie) die klinische Verdachtsdiagnose. Neben einer Vielzahl von Arbeitshilfen, beispielweise einem einheitlichen Protokoll für die Thrombolyse inklusive der Triagekriterien für eine Thrombektomie, sind auch Lehrvideos für die Anwendung der gängigsten Schlaganfallskala (National Institutes of Health Stroke Scale [NIHSS]) oder auch eine Checkliste für den Sekundärtransport in eine (Comprehensive) Stroke Unit hinterlegt. Ein weiterer Schwerpunkt sind die möglichst frühe und gezielte Mobilisierung und Rehabilitation wie auch die Planung der Nachsorge und Weiterführung der Rehabilitation in einer stationären oder ambulanten Versorgungseinrichtung.

### 3. Teilprojekt – stationäre Rehabilitation/Akutnachbehandlung

Ziel ist es, beim Transfer aus dem Akutkrankenhaus eine möglichst nahtlose und zeitnahe rehabilitative Weiterversorgung sicherzustellen. Dazu wurden strukturelle Nachbesserungen sowie eine Prozessoptimierung an den Schnittstellen durchgeführt. Dies betrifft unter anderem die Anmeldung, Terminvergabe und Weitergabe des Therapieberichts an die nächste zuständige Institution. In den Rehabilitationszentren werden interdisziplinär das Therapiekonzept und die Therapieziele definiert und in der wöchentlichen interdisziplinären Evaluierung die Dauer und die weiteren Maßnahmen der Versorgung festgelegt. Werden die Therapieziele erreicht, kann die Planung für die Entlassung aus der stationären Rehabilitation erfolgen. Nun gilt es, die weitere ambulante Therapie inklusive Hilfsmittel zu organisieren und die Sicherstellung der häuslichen Versorgung zu gewährleisten.

### 4. Teilprojekt – ambulante Rehabilitation

Nach der Entlassung aus einer stationären Rehabilitationseinrichtung oder dem Akutkrankenhaus folgt eine Phase mit intensiver ambulanter Therapie in der häuslichen Umgebung des Betroffenen bzw. in entsprechenden Pflegeeinrichtungen. Hier werden Folgetherapie- und Betreuungskonzepte basierend auf der International Classification of Functioning, Disability and Health (ICF) mit den jeweiligen Berufsgruppen sowie mit dem Betroffenen und den Angehörigen vereinbart. Unter Einbindung der Allgemeinmediziner*innen werden die Therapien weiter verordnet. Die Ergebnisqualität aller Maßnahmen wird in einer standardisierten Nachuntersuchung durch Fachärzt*innen für Neurologie 3 Monate nach dem Schlaganfallereignis evaluiert. Anhand dieses Assessments wird entschieden, ob eine weiterführende Therapie zielführend und notwendig ist. Das Konzept der ambulanten Rehabilitation ist voll finanziert und qualitätskontrolliert.

## Teilprojekt Prähospitalphase

Neben regelmäßigen Awareness-Programmen und Presseaktivitäten um das Thema Schlaganfall wurden Plakataktionen in und auf öffentlichen Verkehrsmitteln, Fernseh- und Radioeinschaltungen (Werbespots, Informations‑ und Aufklärungssendungen) und zielgruppenspezifische Informationsveranstaltungen durchgeführt und eigene Websites (https://www.schlaganfall-tirol.at und https://www.schlaganfall-tirol.info) eingerichtet. Um die Akutversorgung durch das Notarzt- und Rettungswesen zu optimieren, wurden im Zuge des Projekts die Abläufe der Notrufbearbeitung, das Erstellen von Einsatzcodes und Diagnosen sowie die Versorgungsintervalle (Versorgungszeit durch Rettungskräfte vor Ort, Prähospitalzeitintervall < 60 min) und medizinische Qualitätsindikatoren (Erhebung des Zeitpunkts des Symptombeginns) angepasst. Aufgrund der komplexen Koordination der unterschiedlichen Blaulichtorganisationen und wegen der Erfahrungen im Rahmen des Lawinenunglücks in Galtür 1999 wurde bereits 2005 eine zentrale Leitstelle geschaffen, die seit 2012 als Leitstelle Tirol die Alarmierung, Koordination, Disposition und Einsatzunterstützung aller Tiroler Einsatzkräfte (mit Ausnahme der Polizei) übernimmt. Bei Eingang des Notrufs wird mittels Kernfragen und eines modifizierten Face-arm-speech-time(FAST)-Tests auf Schlaganfall gescreent und bei Verdacht auf einen Schlaganfall die Austrian Prehospital Stroke Scale (APSS; [5]; Tab. 1) eingesetzt. Hier werden Symptome eines Schlaganfalls abgefragt und anhand der Punktzahl der Schweregrad eingeschätzt.

**Table 1:** 

Item	Frage	Antworten	Maßnahmen	Ergebnis/Ausrückeordnung	APSS
**A**	Bekommt er/sie ausreichend Luft?	Ja	Weiter zu Einleitung	–	–
		Nein	Alarm, dann weiter zu *Einleitung*	Schlaganfall kritisch	–
		Unsicher (kann nicht beurteilt werden)	Weiter zu Einleitung	–	–
		Unbekannt (kein Kontakt/keine Angaben)	Weiter zu Einleitung	–	–
**Einleitung**	Hilfe kommt so schnell wie möglich, bleiben Sie am Telefon. Wir machen gemeinsam einen kurzen Test, um wichtige Informationen für die Rettung zu sammeln	–	Weiter zu *B*	–	–
**B**	Er/sie soll lächeln. War das Lächeln auf beiden Seiten gleich?	Ja, seitengleich	Weiter zu C	Lächeln seitengleich	0
		Nein, unterschiedlich		Lächeln unterschiedlich	1
		Nicht ausgeführt, nicht beurteilbar		Lächeln nicht beurteilt	0
**C**	Er/sie soll folgenden Satz wiederholen: „Die Blumen blühen auf der Wiese.“ War das deutlich und verständlich?	Ja, deutlich und verständlich	Weiter zu D	Sprechen deutlich	0
		Nein, undeutlich oder verwaschen		Sprechen undeutlich	1
		Nein, konnte nicht sprechen		Sprechen nicht möglich	2
		Nicht ausgeführt, nicht beurteilbar		Sprechen nicht beurteilt	0
**D**	Er/sie soll beide Arme ausstrecken und hochhalten. War das auf beiden Seiten gleich?	Ja, seitengleich	Weiter zu E oder Ende	Arme seitengleich	0
		Nein, Unterschied oder Absinken		Arme unterschiedlich	1
		Nein, nur mit einem Arm möglich		Arme nur einseitig beweglich	2
		Nicht ausgeführt, nicht beurteilbar		Arme nicht beurteilt	0
**E**	Er/sie soll beide Beine anheben (hochheben). (WARTEN) Wenn das nicht geht, soll er/sie die Beine anziehen (zur Brust)	Anheben ist beidseits möglich	Weiter zu F	Beine angehoben	0
		Anziehen beidseits ist möglich		Beine angezogen	1
		Keine oder einseitige Bewegung		Beine nicht oder einseitig beweglich	2
		Nicht ausgeführt, nicht beurteilbar		Beine nicht beurteilt	0
**F**	Schaut er/sie starr auf eine Seite? *Ja* → Kann er/sie den Kopf zur anderen Seite drehen? *Nein* → weiter zu „time“	Bewegt Kopf oder kein starrer Blick	Weiter zu G	Kopf bewegt, keine Blickstarre	0
		Kopfbewegung nicht möglich, Blick starr zur Seite		Kopfbewegung nicht möglich, Blickstarre	2
		Nicht ausgeführt, nicht beurteilbar		Kopfbewegung nicht beurteilt	0
**G**	Wann haben diese Beschwerden begonnen? (Exakte Uhrzeit notieren)	Vor weniger als 1 h		t < 1 h	–
		Vor weniger als 2 h		t < 2 h	–
		Vor weniger als 3 h		t < 3 h	–
		Vor weniger als 4 h		t < 4 h	–
		Vor weniger als 5 h		t < 5 h	–
		Vor mehr als 5 h		t > 5 h	–
		Unbekannt oder im Schlaf		t unbekannt	–
**Auswertung der Abfrage**					
**Abfrage APSS**	**Abfrage Zeit**	**Ergebnis**			
≥ 4	< 5 h	Proximaler Schlaganfall			
	< 4 h	Proximaler Schlaganfall			
	< 3 h	Proximaler Schlaganfall			
	< 2 h	Proximaler Schlaganfall			
	< 1 h	Proximaler Schlaganfall			
	Unbekannt	Proximaler Schlaganfall			
≤ 3	< 5 h	Akuter Schlaganfall			
	< 4 h	Akuter Schlaganfall			
	< 3 h	Akuter Schlaganfall			
	< 2 h	Akuter Schlaganfall			
	< 1 h	Akuter Schlaganfall			
	Unbekannt	Akuter Schlaganfall			
≥ 1	≥ 5 h	Schlaganfall			
APSS 0	–	Unklare neurologische Symptome			

Der Schlaganfall wird in Tirol als Notarztindikation eingestuft und eine sorgfältige und schnelle Auswahl eines geeigneten Transportmittels getroffen (Notarzteinsatzfahrzeug bzw. Rettungshubschrauber). Anhand der Einschätzung durch Notärzt*innen vor Ort wird dann das geeignete Zielkrankenhaus anhand eines Entscheidungsbaums ausgewählt. Dieser umfasst

- die Verifizierung der Schlaganfallsymptome,
- die Zeit des Symptombeginns,
- die Blutzuckermessung,
- das Rhythmuselektrokardiogramm,
- die Stabilisierung der Vitalfunktionen,
- den i.v.-Zugang,
- den APSS-Score sowie
- die Beurteilung der Lebensumstände der Patient*innen:
  - Erkrankungen,
  - vorbestehende Bettlägerigkeit,
  - schwere Demenz.

**Figure 3:**
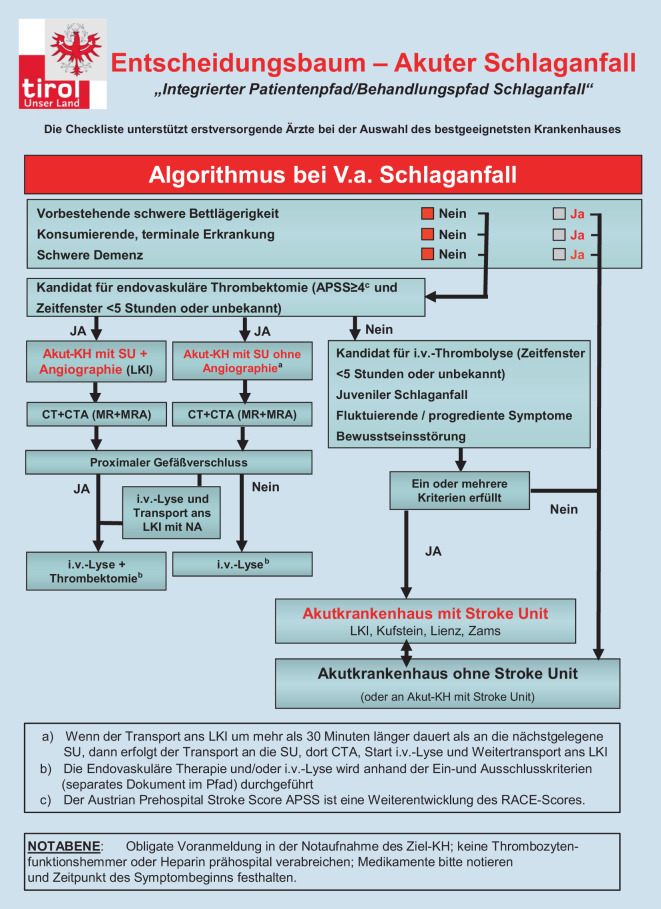

Die standardisierte Vorankündigung an das Zielkrankenhaus gewährleistet die Bündelung der Ressourcen ebendort, um diese auf das Eintreffen vorzubereiten (Abb. 3).

Der Schlaganfall wird in Tirol als Notarztindikation eingestuft

Im Median erreichen 12 der 13 Notarztstützpunkte den Zielerreichungsgrad eines Eintreffens im Krankenhaus innerhalb von 60 min nach Alarmierung (in einem Stützpunkt wurde 2020 ein Median von 64 min berichtet). Je nach Situation und Schweregrad kann es sinnvoll sein, das Schlaganfallzentrum in Innsbruck mit der Möglichkeit zur mechanischen Thrombektomie (Universitätsklinik Innsbruck) direkt anzusteuern. Wenn dieser Transport > 30 min dauert, wird der Primärtransport in das nächstgelegene Akutkrankenhaus mit Stroke Unit zur Bildgebung und zum potenziellen Start einer Thrombolysetherapie erfolgen. Bei Notwendigkeit einer mechanischen Thrombektomie erfolgt nach dem Start einer intravenösen Thrombolysetherapie der Sekundärtransport in die Comprehensive Stroke Unit. Um nicht unnötig Zeit zu verlieren, ist gerade bei Sekundärtransporten eine klare Definition der Zuständigkeiten wichtig.

Die Dokumentation des Symptombeginns ist für die weiterbehandelnden Fachärzt*innen wichtig, insbesondere für die Entscheidung zu den oben genannten Akuttherapiemaßnahmen. Da Schlaganfallpatient*innen in vielen Fällen nicht kommunikationsfähig sind, sind die Kontaktdaten von Angehörigen wichtig, um möglichst alle notwendigen Informationen eruieren zu können. Eine standardisierte Übergabe im Zielkrankenhaus an die weiterbehandelnden Ärzt*innen schließt die Prähospitalphase mit dem Spitalseintritt ab.

## Prähospitale Abschätzung des Schlaganfallschweregrads

Für die rechtzeitige Erkennung von Symptomen eines Schlaganfalls hat sich der Einsatz klinischer Tests bewährt, beispielsweise des FAST-Tests [7], der Cincinnati Prehospital Stroke Scale (CPSS; [8]) oder der Los Angeles Motor Scale (LAMS; [9]). Dabei wird der Schwerpunkt auf motorische Symptome gelegt. Diese beinhalten Elemente des Goldstandardtests der Schlaganfalldiagnostik (NIHSS; [4]). In Österreich wurden die Rettungskräfte in der Anwendung des FAST-Tests geschult, der ab einem erfüllten Parameter als positiv gilt (= Verdacht auf Schlaganfall).

Seit 2015 gibt es mit der mechanischen Thrombektomie eine neue Therapiemöglichkeit [10]. Bei diesem Katheterverfahren können Thromben aus großen Hirnarterien („large vessel occlusion“ [LVO]) mechanisch entfernt werden. Die Technik kann nur in den großen Zentren mit Comprehensive Stroke Units angeboten werden. Mit dieser neuen Therapiemöglichkeit ergeben sich auch Änderungen in der prähospitalen Triage. Da die Schwere des Schlaganfalls stark mit dem Vorliegen eines Hauptgefäßverschlusses korreliert, ist es wichtig, nicht nur die Schlaganfallsymptome, sondern auch die Schwere des Schlaganfalls vor Ort zu evaluieren, um Patient*innen schnellstmöglich der Einrichtung mit Thrombektomiemöglichkeit zuweisen zu können. Dafür wurden neue klinische Scores entwickelt [5], die nicht nur motorische Parameter, sondern auch kortikale Hirnfunktionen berücksichtigen (Tab. 2). Die Blickwendung zum Herd ist das Symptom mit der stärksten Korrelation zum proximalen Gefäßverschluss [11].

**Table 2:** 

	APSS	RACE	G‑FAST	CPSS	NIHSS
Bewusstsein	–	–	–	+	+
Blickwendung	+	+	+	+	+
Hemineglect	–	+	–	–	+
Sehen	–	+	–	–	+
Fazialisparese	+	+	+	–	+
Arme	+	+	+	+	+
Beine	+	+	+	–	+
Ataxie	–	–	–	–	+
Sensibilität	–	–	–	–	+
Sprache	+	+	+	–	+
Sprechstörung	–	–	–	–	+
Punktzahl	0–9	0–9	0–4	0–4	0–42

*APSS* Austrian Prehospital Stroke Scale, *CPSS* Cincinnati Prehospital Stroke Scale, *G‑FAST* „gaze, face, arm, speech, time“, *NIHSS* National Institutes of Health Stroke Scale, *RACE* Rapid Arterial Occlusion Evaluation

Klinische Scores zur Schweregradeinschätzung berücksichtigen neben der Motorik auch kortikale Hirnfunktionen

Vergleiche zwischen den einzelnen Scores gestalten sich schwierig. So konnte eine große Beobachtungsstudie keine signifikanten Unterschiede für die Detektion einer LVO finden [5, 12]. Die APSS wird derzeit in zwei österreichischen Bundesländern in zwei Einsatzvarianten erprobt. Einerseits wird versucht, sie über die Leitstelle bei Erstmeldung (und positivem FAST-Test) abzufragen, andererseits wurden in Wien die Rettungskräfte geschult und Patient*innen bei einem Score über 3 in die Comprehensive Stroke Unit transferiert. Durch die Reduktion der Sekundärtransporte – von der Stroke Unit ins Thrombektomiezentrum – kann dabei die Zeit bis zur Rekanalisierung signifikant reduziert werden.

## Qualitätssicherung – Ergebnisse – Erfolge

Um die implementierten Prozesse überwachen zu können, werden umfangreiche Maßnahmen zur Qualitätssicherung installiert. Diese beinhalten eine verpflichtende Dokumentation des Patient*innenpfads beim Schlaganfall. Um eine lückenlose Datenerfassung zu gewährleisten, wurde tirolweit die International-Classification-of-Diseases(ICD-10)-Codierung der Entlassung, an die in Österreich die Vergütung geknüpft ist, auf I63 und I61 reduziert. Eine Abrechnung ist nur bei vollständig ausgefülltem Datensatz möglich. Diese Dokumentation wird von den jeweiligen Krankenanstalten durchgeführt und zentral durch den Tiroler Gesundheitsfonds (Land Tirol) gesammelt. Eine Plausibilitätskontrolle der Daten sowie eine Kontrolle der Vollständigkeit wird quartalsweise durch den Abgleich mit den Informationen aus der leistungsorientierten Krankenhausfinanzierung durchgeführt, fehlende Daten werden nachgefordert bzw. es wird um Bereinigung gebeten (Tab. 3).

**Table 3:** 

Zeitmanagement	Klinische Präsentation	Behandlung/weitere Diagnostik	Risikofaktoren
Datum/Uhrzeit Ereignis	NIHSS [4] bei Aufnahme	Thrombolyse	Diabetes
Datum/Zeit Krankenhausaufnahme	mRS [13] vor dem Schlaganfall	Echokardiographie	Vorinsult
Zutransport	–	Dysphagietestung	Orale Antikoagulation
Zeitpunkt Bildgebung	–	Physiotherapie, Ergotherapie, Logopädie	Vorhofflimmern
Zeitpunkt Gefäßdarstellung	–	Transfer Rehabilitationseinrichtung/Pflegeheim	Komplikationen

*mRS* modifizierte Rankin-Skala, *NIHSS* National Institutes of Health Stroke Scale

Zur Qualitätssicherung und -verbesserung wird zusätzlich einmal pro Jahr ein Bericht seitens des Tiroler Gesundheitsfonds erstellt, der einen anonymen Vergleich der Krankenanstalten ermöglicht und sowohl im Expert*innengremium als auch in persönlichen Gesprächen mit der entsprechenden Krankenanstalt diskutiert wird. Die Prozessabläufe und die Qualität der Schlaganfallversorgung werden auf Ebene der Krankenanstalten und zusätzlich auf Ebene der Wohnbezirke ausgewertet und verglichen. So können auch regionale Probleme aufgezeigt werden, beispielsweise lange Antransportzeiten oder eine Unterversorgung mit Thrombolysebehandlung oder Rehabilitation. Regelmäßige Treffen der Projektbeteiligten in den verschiedenen Krankenanstalten sollen Versorgungsprobleme aufzeigen und Verbesserungen des Behandlungsalgorithmus einleiten. So konnten beispielsweise in den letzten Jahren in einzelnen Regionen höhere Sekundärtransportraten von akuten Schlaganfallpatient*innen, die nicht direkt an ein Lysezentrum transportiert wurden, beobachtet werden. Nach Evaluation der Fälle wurden punktuell Nachschulungen im Prähospitalbereich angeboten. Im Folgenden werden einzelne Ergebnisse des Tiroler Schlaganfallpfads erläutert.

### Thrombolyserate und Outcome nach Implementierung des Schlaganfallpfads

Im Jahr 2007 betrug die Thrombolyserate beim ischämischen Schlaganfall in Tirol flächendeckend 6,6 %. Nach Implementierung des Tiroler Schlaganfallpfads verdoppelte sie sich nahezu (12,9 %). In den folgenden drei Jahren stieg sie weiter auf 16,8 % [2]. Ursächlich für den Anstieg der Thrombolyserate sind die bessere Vernetzung aller Berufsgruppen sowie eine Homogenisierung der Thrombolyserate in ganz Tirol. Auch in anderen österreichischen Bundesländern, die einen vergleichbaren Schlaganfallpfad implementiert haben, ist die Thrombolyserate deutlich angestiegen [2].

Das klinische Outcome nach 3 Monaten hat sich nach Implementierung des Schlaganfallpfads ebenso deutlich verbessert. Während noch im Jahr 2010 etwa 40 % der Schlaganfallpatient*innen ein exzellentes funktionelles Outcome (modifizierte Rankin-Scale [mRS] 0–1, keine von Patient*innen bemerkbare Defizite) nach 3 Monaten erreichten, waren es im Jahr 2013 schon mehr als 50 %. Ähnlich verhalten sich die Zahlen für ein gutes funktionelles Outcome (mRS 0–2, allenfalls geringe Defizite) mit 56 % im Jahr 2010 gegenüber 66 % im Jahr 2013. Auch eine Homogenisierung des klinischen Outcomes bei Schlaganfallpatient*innen über alle Bezirke im Bundesland Tirol wurde durch den Schlaganfallpfad erreicht [2].

Weitere Erfolge sind eine deutliche Verbesserung der logopädischen Betreuung von Schlaganfallpatient*innen, ein obligates Dysphagiescreening und eine deutliche Abnahme von Aspirationspneumonien von initial knapp 10 % auf nun mehr 2,5 %. Diagnostische Abläufe wurden wesentlich beschleunigt. Knapp 80 % aller Schlaganfallpatient*innen erhalten die erste Bildgebung innerhalb einer Stunde, knapp 60 % erhalten eine transösophageale oder transthorakale Echokardiographie, 90 % eine Ultraschalluntersuchung der hirnzuführenden Gefäße [14]. Gerade die Coronavirus-disease-2019(COVID-19)-Pandemie mit den begleitenden Lockdown-Maßnahmen stellt eine Herausforderung an alle Bereiche des Gesundheitssystems dar. Besonders in diesem Setting sind klare Prozessabläufe und Zuständigkeiten sowie eine gut etablierte Kommunikation zwischen den beteiligten Disziplinen essenziell. Die ausgewerteten Daten des Jahres 2020 zeigen, dass der Versorgungsprozess über den Schlaganfallpfad äußerst stabil ist und die Prozessabläufe durch die Pandemie nicht beeinträchtigt wurden (Publikation in Vorbereitung).

## Diskussion

**Figure 4:**
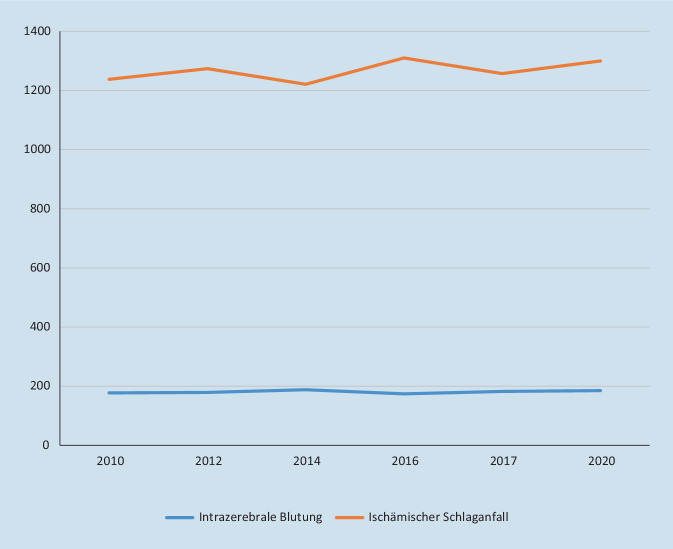

Der Schlaganfallpfad Tirol konnte ein standardisiertes Konzept etablieren, das die gesamte Schlaganfallversorgungskette optimiert und eine stärkere Vernetzung aller beteiligten Berufsgruppen und Sektoren gefördert hat. Nach Implementierung hat sich die Thrombolyserate fast verdoppelt und die Rate des guten funktionellen Outcomes ist im Laufe der Jahre angestiegen. Die Anzahl der Patient*innen im Schlaganfallpfad Tirol ist in den letzten Jahren konstant geblieben bzw. gering gestiegen (Abb. 4). Gemäß den Leitlinien der American Stroke Association und der American Heart Association liefert das Projekt eine Evidenzlevel-IB-Empfehlung für evidenzbasierten Gebrauch der Thrombolysetherapie und gute Outcomes nach Schlaganfall in Versorgungsprogrammen [14]. In den letzten Jahren wurden viele Nachsorgekonzepte nach ischämischem Schlaganfall umgesetzt. Der europäische Aktionsplan Schlaganfall 2018–2030 empfiehlt die Etablierung umfassender Schlaganfallpfade in allen Regionen und Ländern Europas [15]. Neben dem Schlaganfallpfad Tirol mit der ambulanten Rehabilitation gibt es keine qualitätsgesicherten publizierten Schlaganfallnachsorgeprogramme mit multimodalen Komponenten, welche die gesamte Versorgungskette umfassen [16].

Das Konzept der mobilen Stroke Units ist in komplexen geografischen Lagen keine Option

Ein anderes Konzept der Akutversorgung sind die sogenannten mobilen Stroke Units. Hier werden mobile Computertomographen sowie das nötige Material und Medikamente mit dem Fahrzeug zum Patienten transportiert und die Akutversorgung mit potenzieller Thrombolyse extramural durchgeführt [17]. Das Konzept konnte bisher jedoch nur in urbanen Regionen überzeugen und ist in komplexen geografischen Lagen wie in Tirol keine Option. Prähospitalscores wie der FAST-Test und die APSS können eine bessere Organisation der Rettungskette ermöglichen und sollten flächendeckend eingesetzt werden.

## Fazit für die Praxis

- Mit dem Schlaganfallpfad Tirol konnte ein standardisiertes Konzept etabliert werden, das die gesamte Schlaganfallversorgungskette optimiert und eine stärkere Vernetzung aller beteiligten Berufsgruppen und Sektoren gefördert hat.
- Das Konzept der integrierten Versorgung mit Optimierung der Versorgungsprozesse hat zu einer signifikanten Verbesserung der Ergebnisqualität 3 Monate nach dem akuten Schlaganfallereignis geführt.
- Nach Implementierung hat sich die Thrombolyserate fast verdoppelt und die Rate des guten funktionellen Outcomes ist angestiegen.
- Für eine schnelle und effektive Rettungskette werden Prähospitalscores empfohlen.
